# Phylogenetic analysis of the complete chloroplast genome of *Gracilaria vermiculophylla*

**DOI:** 10.1080/23802359.2020.1765708

**Published:** 2020-05-22

**Authors:** Yan Li, Hongbin Han, Xiaojun Ma

**Affiliations:** a Key Laboratory of Marine Eco-Environmental Science and Technology, First Institute of Oceanography, the Ministry of Natural Resources, Qingdao, China; b Laboratory of Marine Ecology and Environmental Science, Pilot National Laboratory for Marine Science and Technology (Qingdao), Qingdao, China

**Keywords:** Chloroplast genome, *Gracilaria vermiculophylla*, phylogenetic analysis, *Gracilaria tenuistipitata*, *Gracilaria changii*

## Abstract

In this study, we sequenced and annotated the complete chloroplast genome of *Gracilaria vermiculophylla* (GenBank accession number: MN853882). The total length of the chloroplast genome of *G. vermiculophylla* is 180,262 bp, including 191 protein-encoding genes, 30 tRNA genes and 3 rRNA genes. The phylogenetic tree, which is based on core genes, shows that *G. vermiculophylla* clustered into the *Gracilaria* clade and has close genetic relationships with algae *Gracilaria tenuistipitata* and *Gracilaria changii*. These data will enable a better understanding of the phylogenetic status of *G. vermiculophylla*.

*Gracilaria vermiculophylla* belongs to the phylum Rhodophyta, class Florrdeophyceae, order Gracilariaceae, and family *Gracilaria* (Zeng et al. 2005). *G. vermiculophylla* was originally described in Japan and has a native distribution in the East of Asia. During the past three-to-four decades, this seaweed has successfully invaded many coastal habitats around the globe (Rueness 2005). *Gracilaria vermiculophylla* is one of the most important raw materials for the agar industry (Zhang et al. 2005) and is also a good heavy metal adsorption alga, which can significantly improve the water environment (Riosmena et al. 2010). With the recent development of genomics, an increasing number of chloroplast genomes of macroalgae have been sequenced and published (Leliaert and Lopez-Bautista et al. 2015; Melton et al., 2015; Cai et al. 2017); however, the chloroplast genome of *G. vermiculophylla,* an important economic alga, has not previously been reported. Therefore, we determined the complete chloroplast genome sequence of *G. vermiculophylla*.

*Gracilaria vermiculophylla* was collected from the Qinhunangdao coastal area (39°49′54.69″N, 119°31′30.50″E) and cultured in the laboratory with VSE media at 20 °C under a light intensity of 100 μmol^−2^ s^−1^ (Zhou et al. 2016a, 2016b). The specimens were preserved at the Marine Ecology Research Center of the First Institute Oceanography, Ministry of Natural Resources in Qingdao (Accession number: ZJL06).

The shape of the chloroplast genome of *G. vermiculophylla* is a double-stranded closed loop. The GenBank accession number is MN853882. The overall base composition of the *G. vermiculophylla* chloroplast genome is 25.99% A, 25.85% T, 24.08% C, and 24.08% G. The complete chloroplast genome is 180,262 bps long. There are 191 protein-coding genes in the genome, including 30 *ycf* genes, 27 *rpl* genes, 19 *rps* genes, 18 *psb* genes, 11 *psa* genes, 9 *pet* genes, 8 *atp* genes, 5 *apc* genes, 4 *rpo* genes, 3 *acc*, *sec* and *cpc* genes, 2 *ccs*, *cpe*, *dna*, *ilv*, *inf*, *odp*, *rbc*, *suf*, *thi*, and *trp* genes. In addition, there are some genes that are contained only once in the genome, such as *acp*, *arg*, *bas*, *car*, *cbb*, *cem*, *chl*, *clp*, *dfr*, *dsb*, *fab*, *ftr*, *fts*, *glt*, *gro*, *lys*, *moe*, *nbl*, *ntc*, *omp*, *pbs*, *pgm*, *pre*, *rne*, and *rnz*. In addition, the chloroplast genome contains 30 tRNA genes and 3 rRNA genes (*rns*, *rrn5*, and *rnl*), which are all non-coding. All the coding genes begin with ATG, except for *infC*, *psaF*, *rbcS*, and *rps8,* which begin with GTG. The termination codons for *apcD*, *dsbD*, *odpB*, *petB*, *petL*, *psaC*, *psaD*, *psbA*, *psbJ*, *psbK*, *rpl27*, *rpl34*, *rps14*, *rps2*, *rps3*, *rps5*, and *trpG* are TAG. However, *acpP*, *apcD*, *atpF*, *lysR*, *petF*, *psaF*, *psbN*, *rpl33*, *rpl5*, *rpoC1*, *rps16*, *rps17*, *ycf29*, *ycf52*, and *ycf58* terminate with TGA. The remaining 159 genes end with TAA.

This is the first record of the chloroplast genome of *G. vermiculophylla*. The phylogenetic relationship of *G. vermiculophylla* with other species was estimated using a phylogenetic tree that was constructed based on the core genes of *G. vermiculophylla* as well as 24 other species. The phylogenetic tree (Figure 1) shows that *G. vermiculophylla* clustered into the *Grateloupia* clade and has close genetic relationships with algae *Grateloupia filicina* and *Grateloupia taiwanensis.*

**Figure 1.**
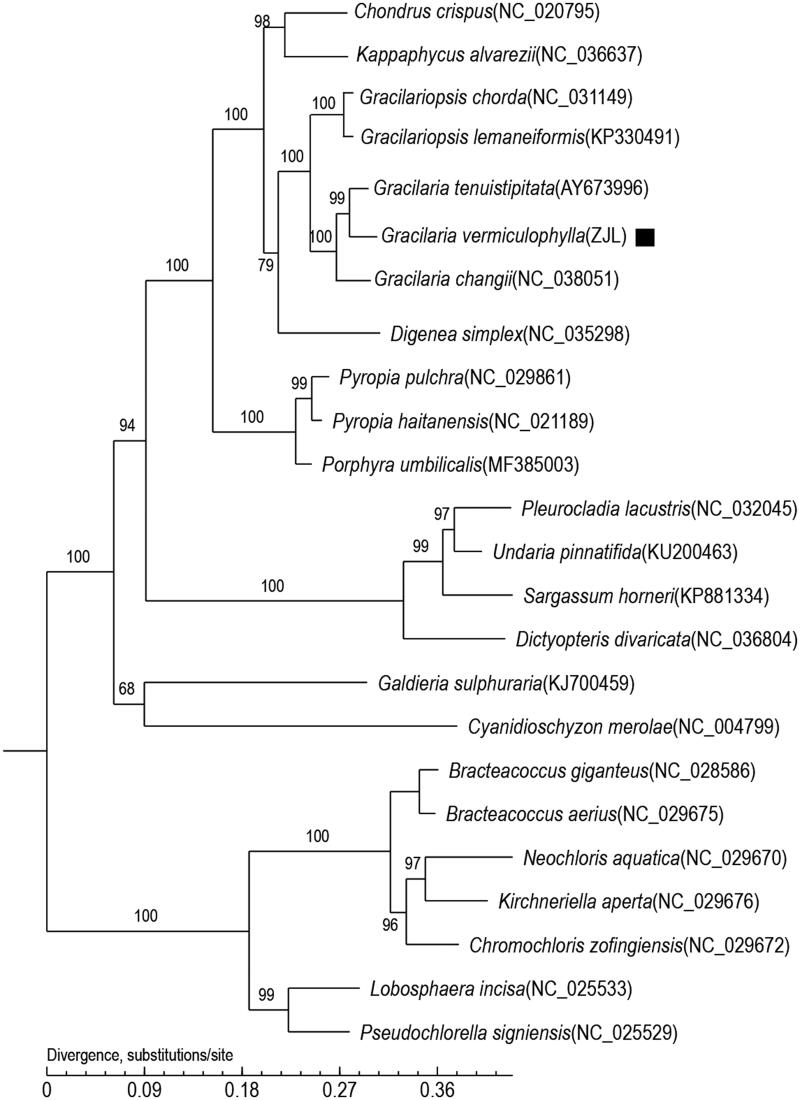
Maximum-likelihood (ML) tree based on the complete chloroplast genome sequences of 24 species. The numbers on the branches are bootstrap values.

## Data Availability

The data that support the findings of this study are openly available in NCBI GenBank database at https://www.ncbi.nlm.nih.gov/ with the accession number is MN853882.
